# Activation of Cell Cycle Arrest and Apoptosis by the Proto-Oncogene Pim-2

**DOI:** 10.1371/journal.pone.0034736

**Published:** 2012-04-10

**Authors:** Daphna Levy, Ateret Davidovich, Shahar Zirkin, Yulia Frug, Amos M. Cohen, Sara Shalom, Jeremy Don

**Affiliations:** 1 The Mina and Everard Goodman Faculty of Life Sciences, Bar-Ilan University, Ramat-Gan, Israel; 2 Hemato-Oncology Unit, Davidoff Center, Rabin Medical Center, Petach-Tikva, Israel; Florida International University, United States of America

## Abstract

Potent survival effects have been ascribed to the serine/threonine kinase proto-oncogene PIM-2. Elevated levels of PIM-2 are associated with various malignancies. In human cells, a single Pim-2 transcript gives rise mainly to two protein isoforms (34, 41 kDa) that share an identical catalytic site but differ at their N-terminus, due to in-frame alternative translation initiation sites. In this study we observed that the 34 kDa PIM-2 isoform has differential nuclear and cytoplasmic forms in all tested cell lines, suggesting a possible role for the balance between these forms for PIM-2's function. To further study the cellular role of the 34 kDa isoform of PIM-2, an N-terminally HA-tagged form of this isoform was transiently expressed in HeLa cells. Surprisingly, this resulted in increased level of G1 arrested cells, as well as of apoptotic cells. These effects could not be obtained by a Flag-tagged form of the 41 kDa isoform. The G1 arrest and apoptotic effects were associated with an increase in T14/Y15 phosphorylation of CDK2 and proteasom-dependent down-regulation of CDC25A, as well as with up-regulation of p57, E2F-1, and p73. No such effects were obtained upon over-expression of a kinase-dead form of the HA-tagged 34 kDa PIM-2. By either using a dominant negative form of p73, or by over-expressing the 34 kDa PIM-2 in p73-silenced cells, we demonstrated that these effects were p73-dependent. These results demonstrate that while PIM-2 can function as a potent survival factor, it can, under certain circumstances, exhibit pro-apoptotic effects as well.

## Introduction

Pim-2 is a member of a serine threonine kinase family of proto-oncogenes, which include also Pim-1 and Pim-3. The family was identified as a common proviral insertion site of MuLV (Moloney murine leukemia virus) in T and B cell lymphomas in mice [1]–[3]. Transgenic mice over-expressing either Pim-1 or Pim-2 are predisposed to T cell lymphomas, whereas both Pim-1 and Pim-2 act synergistically with c-Myc to accelerate development of B-cell tumors [2], [4]–[6]. Pim-1 or Pim-2 deficient mice show no *in-vivo* abnormalities [7]–[8]. However, Pim-1-Pim-2 double knockout, or even more so, a triple knockout of all three Pim genes, causes a mild phenotype of reduced body size, impaired proliferation of hematopoietic cells in response to a variety of growth factors, and an effect on the cell cycle entry of peripheral T cells in response to IL-2 and TCR activation [9].

In humans, increased levels of PIM-2 were described in different hematological malignancies. For example, in Non-Hodgkin Lymphoma (NHL) and in Chronic Lymphocytic Leukemia (CLL), significant up-regulation of PIM-2 was observed [10]. Moreover, PIM-2 levels in CLL correlate with the clinical stage of the disease and to the lymphocyte doubling time, and it was suggested that PIM-2 contributes to the lymphomagenesis by functioning as a survival factor [10]. Pim-1 and Pim-2 were also reported to be required for efficient pre-B-cell transformation by the Abl oncogene [11]. In addition, elevated levels of PIM-2 were associated with severe clinical and pathological symptoms in prostate cancer, and hence with poor prognosis [12]–[13]. Most documented substrates of PIM-2, so far, function as anti-apoptotic/survival factors upon phosphorylation. In the murine IL-3-dependent pro-B cell line, FL5.12, constitutive expression of Pim-2 confers dose-dependent resistance to apoptosis following growth factor withdrawal, and the survival promoting effect of PIM-2 is dependent on its catalytic activity [14]. It was suggested that PIM-2 (as well as Akt) contributes to the phosphorylation of the pro-apoptotic factor 4E-BP1 on Ser 65, abrogating its inhibitory interaction with elF-4e, and hence enabling the formation of an active translational initiation complex which is linked to increased apoptotic resistance [14]. PIM-2 can also act as a pro-survival kinase by phosphorylating BAD on Ser 112 [15]–[16], preventing its interaction with Bcl-X_L_ and thus inhibiting its pro-apoptotic activity [16]. Cot, the IκB kinase activator was also reported as a pro-survival target of PIM-2 [17]. Additional pro-survival targets of PIM-2 are the eukaryotic initiation factor eIF4B and the apoptosis inhibitor API-5, both involved in cell growth and survival [18]. Finally, PIM-2 can enhance the transforming activity of c-Myc by phosphorylating it at Ser329 and thus stabilizing the protein and increasing its transcriptional activity [19], and promote tumorigenesis by down-regulating expression of the cell cycle inhibitor p27^kip1^ at both the transcriptional and post-translational levels [20]. These studies, and others, have promoted the notion that targeting PIM-2's kinase function could have beneficial therapeutic effects, as has indeed been shown regarding CLL [21].

In mice, a single Pim-2 transcript gives rise to three isoforms of the protein (34, 37 and 40 kDa) that share an identical catalytic site but differ at their N-termini, due to in-frame alternative translation initiation sites [8], [15]. In human cells, however, only two such isoforms (34 and 41 kDa), were evident [22].

In this study we observed that the 34 kDa PIM-2 isoform has differential nuclear and cytoplasmic forms in all tested cell lines, suggesting a possible role for the balance between these forms for PIM-2's function. To further study the cellular role of the 34 kDa isoform of PIM-2, we over-expressed a HA-tagged form of this isoform of PIM-2 in HeLa cells, and surprisingly found that this isoform, but not a Flag-tagged 41 kDa isoform, led to cell cycle arrest at G1 and to increased apoptosis. These effects were associated with increased Thr14/Tyr15 phosphorylation of CDK2, decreased levels of nuclear CDC25A, as well as increased levels of E2F-1, p73 and p57. Co-expression of PIM-2 with a dominant negative form of p73, as well as over-expressing the 34 kDa PIM-2 in p73-silenced cells, abrogated the cell cycle arrest and pro-apoptotic effects, pointing at p73 as a central link in the pathway leading to this phenotype. These results suggest that PIM-2 can play a dual role. Under certain circumstances it increases cell survival, while under different conditions it might act to promote cell cycle arrest and apoptosis.

## Results

### Intracellular distribution of PIM-2 in various cell lines

**Figure 1:**
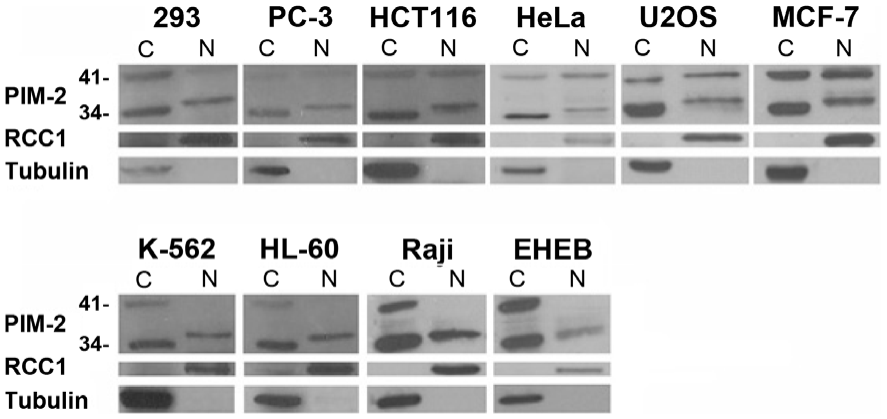
Western blot analysis of cytoplasmic(C) and nuclear(N) expression of PIM-2 in various cell lines. Nuclear or cytoplasmic proteins (50 µg) were separated on a 15% SDS-PAGE. Blots were reacted with anti PIM-2 antibodies as primary antibody and HRP conjugated anti rabbit IgG secondary antibody. The membranes were stripped twice and reacted once with anti RCC1 antibody as control for nuclear proteins and once with anti β-tubulin as control for cytoplasmic proteins.

The Pim kinases were defined as cytoplasmic survival kinases that phosphorylate substrates that contribute to cell survival [23]. Nonetheless, it was reported that in Burkitt's lymphoman, nuclear localization of PIM-1 was required for its biological effect [24]. Furthetmore, PIM-1 performed nuclear localization in the human myocardium upon infarction injury, inducing a cardioprotective effect [25]. So, our first attempt was to determine PIM-2's intracellular distribution in various solid tissue and hematological human cell lines (HEK293, PC-3, HCT116, HeLa, U2OS, MCF-7, and K562, HL-60, Raji and EHEB, respectively). As expected, two isoforms of about 34 kDa and 41 kDa were consistently observed in the cytoplasmic fraction of all cell lines. As to the nuclear fraction, an apparent signal of the 41 kDa isoform appeared in all solid-tissue cell lines, but much less so in the hematological cell lines. A third predominant form, with a slightly slower mobility than the cytoplasmic 34 kDa isoform, appeared exclusively in the nuclear fraction of all cell lines (Fig. 1). These results suggest that PIM-2 might have a distinct nuclear function and that some variation in structure or modification between the cytoplasmic and nuclear forms may determine their locations. Nuclear localization of PIM-2 was also verified immunocytochemically in all tested cell lines (Fig. S1).

### Cytoplasmic and nuclear over-expression of the 34 kDa isoform of PIM-2 in HeLa cells

**Figure 2:**
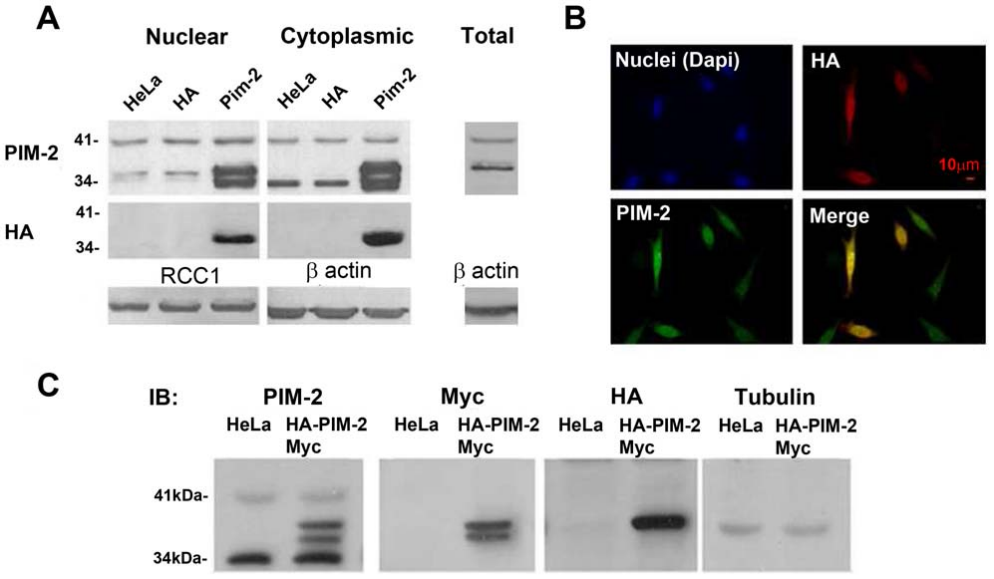
Over-expression of the 34 kDa isoform of the human PIM-2 protein in HeLa cells. (**A**) Nuclear and cytoplasmic protein extracts (50 µg) from cells over-expressing HA-PIM-2 (Pim-2), control cells transfected with a HA vector (HA) or untreated control cells (HeLa), were analyzed by Western blotting using antibodies against either the PIM-2 protein or the HA-tag. For equal loading reference, the blot was stripped and re-probed with antibodies against β-actin (cytoplasmic fractions) or RCC1 (nuclear fractions). (**B**) Immunocytochemical analysis of HeLa cells transiently transfected with the HA-Pim-2 encoding plasmid, using either rabbit anti-PIM-2 antibodies (PIM-2) or mouse anti-HA antibodies (HA). Alexa 488 conjugated anti-rabbit (green) and Alexa 594 conjugated anti-mouse (red) secondary antibodies were used for PIM-2 and HA detection, respectively. In right lower panel PIM-2 and HA signals are merged. Nuclei were stained with Dapi (blue). Bar represents 10 µm. (**C**) Total protein extracts (50 µg) from cells over-expressing the 34 kDa isoform of PIM-2 tagged with the HA-tag at its N-terminus and with the Myc-His-tag at its C-terminus (HA-PIM-2-Myc), or from untreated control cells (HeLa), were analyzed by Western blotting, using antibodies against the PIM-2 protein (PIM-2). The blot was stripped and re-probed with antibodies against the Myc-tag (Myc), stripped again and re-probed with antibodies against the HA-tag. For equal loading reference, the blot was stripped once again and re-probed with antibodies against tubulin.

To start to elucidate the function of the 34 kDa isoform of PIM-2, primarily in solid tissue cells, we transiently transfected HeLa cells with a plasmid encoding the 34 kDa isoform of PIM-2 tagged with the HA tag at its N-terminus. After 48 hours, cytoplasmic and nuclear proteins from HA-PIM-2 expressing cells, as well as from control cells (transfected with an empty HA plasmid or untreated), were analyzed by Western blotting, using either anti PIM-2 or anti HA antibodies. As can be seen in Figure 2A, using the PIM-2 antibodies we could detect an apparent increase in the amount of not only the 34 kDa isoform, but of the slightly slower running form as well, in both the cytoplasm and the nucleus, verifying that the slower migrating form indeed relates to the 34 kDa isoform of PIM-2. No change was observed in the 41 kDa isoform. Interestingly, only the higher, slower migrating, form of the 34 kDa isoform, but not the lower 34 kDa form, could be detected by anti HA antibodies, in both the cytoplasmic and nuclear fractions (Fig. 2A). Immunocytochemical verification of the expression of the recombinant HA-PIM-2 in the transfected cells also pointed at intense nuclear localization of the recombinant protein (Fig. 2B). It is noteworthy, however, that the 34 kDa nuclear form of PIM-2 could readily be detected in the nuclear proteins due to its amplification in the nuclear protein lysate. In total protein lysates from control cells, the amount of this slower mobility form (nuclear form) is considerably smaller then that of the higher mobility form (cytoplasmic form) and it can be detected in high resolution gels after rather long running time (Fig. 2A).

Assuming that the two HA-PIM-2 forms obtained correspond to the endogenous cytoplasmic and nuclear forms, both HA-tagged forms were analyzed, using MassSpectrometry, to determine whether one is a post-translationally modified form compared to the other. We found that while both forms are indeed PIM-2, as expected, the higher (slower migrating) form is not post-translationally modified, compared to the lower 34 kDa form, by either phosphorylation, glycosylation, ubiquitilation, sumoylation or acetylation. The lack of phosphorylation and glycosylation was further verified by phosphatase and glycosidase assays, respectively (Fig. S2). However, the 4 most N-terminal amino acids of the lower 34 kDa form of PIM-2, together with the HA-tag, could not be detected by the MassSpecrometric analysis, whereas the 34 kDa N-terminus, containing the HA-tag, could readily be detected in the higher form. Moreover, when we repeated the transient transfection experiments with a plasmid encoding the 34 kDa isoform of PIM-2 tagged with the HA-tag at its N-terminus and with a Myc-His-tag at its C-terminus, two recombinant forms were detected by the Myc antibody whereas only the higher (slower migrating) form could be detected by the HA antibody (Fig. 2C). Both recombinant forms, together with the endogenous PIM-2, were detected in total protein lysates by the PIM-2 antibody. These results suggest that the two 34 kDa forms differ by a modification that involves truncation of the peptide's N-terminus, and that this modification might determine, in an as of yet unknown way, the intracellular location.

### PIM-2 induces cell cycle arrest and increased apoptosis in HeLa cells

**Figure 3:**
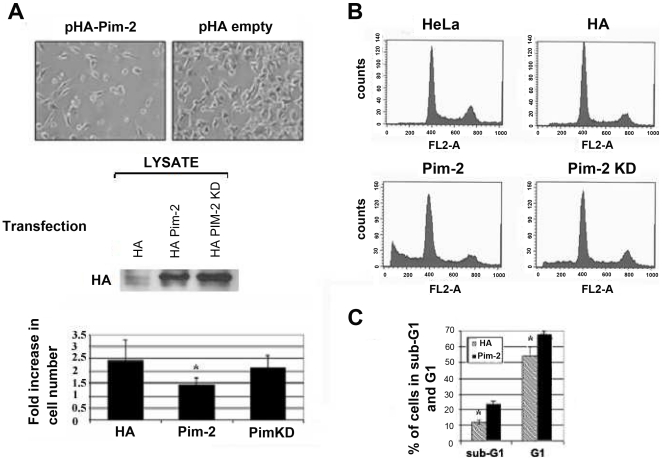
PIM-2's overexpression in HeLa cells promotes G1 arrest and increases apoptosis. (**A**) Upper panel - Light microscope images (×40) of cells 48 hours after transfecting equal amounts of cells with either HA-Pim-2 vector (pHA-Pim2) or empty control vector (pHA-empty), and under identical culture conditions. Middle panel – Western blot analysis, using anti HA antibodies, showing the relative amounts of either HA-PIM-2 or HA-PIM-2 KD in the tested cells. Lower panel - Fold increase in cell number calculated 48 hours after transfecting equal amounts of cells with either the HA-Pim2 plasmid (Pim-2), an empty HA control vector (HA) or with a kinase dead form of Pim-2 (PimKD). Data shown are the average of four independent experiments. Asterisk represents statistically significant differences (p<0.05). (**B**) FACS analysis of cell cycle distribution of PI stained cells 48 hours after transfection with either HA-Pim2 plasmid (Pim-2), HA-Pim2 kinase-dead plasmid (Pim-2 KD), control empty vector (HA) or untreated cells (HeLa). (**C**) A comparison between the average percentage of cells (four independent experiments) at the sub-G1 and G1 phases, in Pim-2 expressing cells versus HA control cells (Asterisks represents statistically significant differences p<0.014 and p<0.007, respectively).

Given the anti-apoptotic function attributed to PIM-2, we expected that PIM-2 would promote survival upon over-expression in HeLa cells. Surprisingly, we found that 48–72 hours after transfection of equal amounts of cells with either the HA-Pim2 plasmid (Pim-2) or with an empty HA plasmid as control (HA), the HA-Pim-2 expressing cells exhibited significant growth arrest at the G1 phase (p<0.007) and increased percentage of apoptotic cells (p<0.014), compared to control cells (Fig. 3A–C). The increased rate of apoptosis in PIM-2 over-expressing cells was further verified by an annexin-V assay (not shown). Cell density in HA control cells after 48 hours was twice as that of the PIM-2 expressing cells (p<0.05, Fig. 3A). However, over-expressing equal amounts of a HA-tagged kinase-dead form of the 34 kDa PIM-2 (PimKD) in HeLa cells induced only a minor and statistically insignificant reduction in cell growth, and apoptotic rates were comparable to the HA control (Fig. 3A–B). Silencing Pim-2 in HeLa cells via siRNA (Fig. S3A), did not significantly affect cell growth or survival. On the contrary, the siRNA transfection reagent had a somewhat toxic effect on the culture and silencing of Pim-2 seemed to rescue the cells from this toxic effect (Fig. S3B). An *in-vitro* kinase assay was performed to verify that the recombinant HA-PIM-2 is indeed kinase active, using a recombinant Bad as a substrate. As shown in Fig. S4, HA-PIM-2 effectively phosphorylated Bad whereas only trace phosphorylative activity was obtained by the HA-PIM-2-KD. These results suggest that the observed inhibition of cell growth depends mostly on the kinase activity of PIM-2.

To test whether the cell growth retardation and increased apoptosis upon over-expression of the 34 kDa PIM-2 isoform, is unique to HeLa cells, we constructed a stable Tet-on-inducible system in U2OS cells (human osteosarcoma) in which expression of either HA-PIM-2, or HA-PIM-2KD are activated by Doxycycline (Dox.) in a dose-dependent manner (Fig. S5A). We found that cell death increased as the Tet-on PIM-2 culture was exposed to increasing Dox. Concentrations (for 96 h), as determined by the MTT assay. No such increased death was seen in U2OS Tet-on control cells. PIM-2KD cultures exhibited a mild decrease in cell survival compared to the PIM-2 expressing cells (Fig. S5B). Accordingly, cell cycle analysis, after exposure to Dox. (2 µg/ml) for 96 h, revealed increased amount of sub-G1/apoptotic cells in PIM-2 expressing cells compared to control cultures. Interestingly, an S-phase arrest, rather then G1 arrest, was seen in the PIM-2 cultures in these experiments. These results, and similar results we obtained also with B16 mouse melanoma cells (not shown), suggest that this phenomenon is not restricted to HeLa cells but rather of a more general nature.

To test whether both isoformes of PIM-2 can activate the G1 arrest and apoptotic effects, we transiently over-expressed a C-terminally Flag-tagged form of the 41 kDa isoform, in both HeLa and U2OS cells, and no such effect could be seen up to 96 h after transfection. This was even more substantiated when we silenced PIM-2 in U2OS cells and then over-expressed either the 34 kDa or the 41 kDa isoformes in the silenced cells (Fig. S6A), monitoring the sub-G1 phase of the cells 72 h after transfection. As can be seen in Fig. S6B–C, over-expression of the 34 kDa isoform in the Pim-2-silenced cells had an even intensified apoptotic effect compared to un-silenced cells, treated with non-specific shRNA, whereas over-expression of the 41 kDa isoform in the silenced cells had no effect. These results point to differential effects of the two PIM-2 isoforms on the cell, and that only the 34 kDa isoform can activate a pro-apoptotic effect.

### PIM-2 over-expression is associated with elevated levels of p57 and T14/Y15 phosphorylation of CDK2 and reduced levels of CDC25A

**Figure 4:**
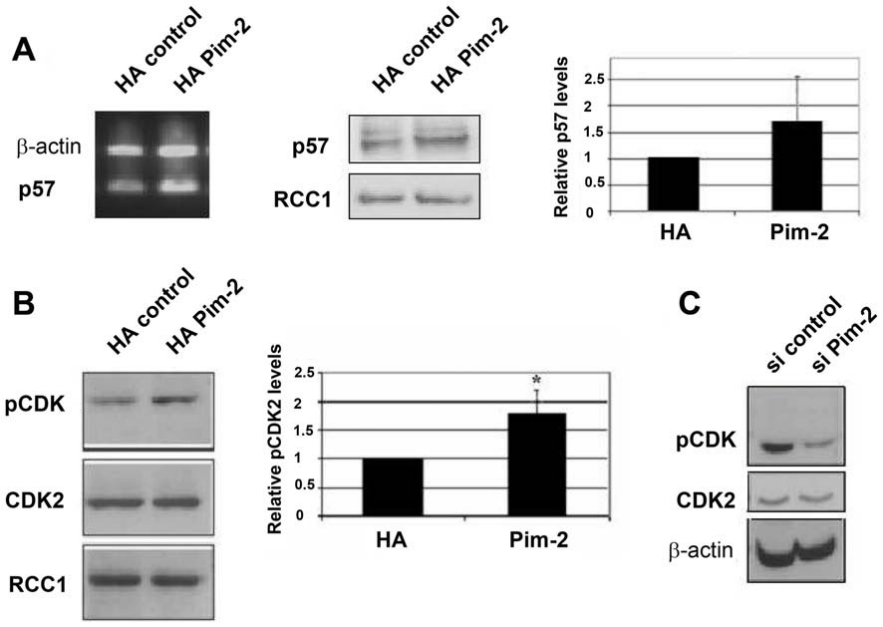
p57 expression and T14/Y15 phosphorylation on CDK2 in HeLa cells over-expressing the 34 kDa PIM-2 isoform. (**A**) RT-PCR analysis of p57 transcripts in control cells (HA control) and in Pim2 over-expressing cells (HA Pim-2). Actin specific primers were used as reference for equal loading. Western blot analysis was used to evaluate the p57 protein in nuclear extracts (40 µg) from HA control cells and from cells over-expressing Pim-2 (HA Pim-2). Anti-RCC1 antibodies were used as reference for equal loading. The average relative level of p57 in Pim-2 over-expressing cells (Pim-2) compared to HA control cells (HA) is depicted in the right panel. Comparison was based on densitometric analysis of p57 signals normalized according to the RCC1 signal (average of three independent experiments). Average level of p57 in control cells was determined as 1. Differences were just above the statistically significance of p<0.05. (**B**) Western blot analysis of phosphorylated CDK2 on T14/Y15 (pCDK2) in nuclear extracts from cells over-expressing Pim-2 (HA Pim-2) and HA control cells, using phospho T14/Y15 specific antibodies. Blots were stripped and reprobed once with CDK2 specific antibodies and then once again with RCC1 antibodies as reference for equal loading. The average relative level of pCDK2 in Pim-2 over-expressing cells (Pim-2) compared to HA control cells (HA) is depicted in the right panel. Comparison was based on densitometric analysis of pCDK2 signals normalized according to the RCC1 signal (average of three independent experiments). Average level of pCDK2 in control cells was determined as 1. Asterisk represents statistically significant differences (p<0.04) (**C**) Western blot analysis of pCDK2 in total protein extracts (40 µg) from Pim-2 silenced cells, via siRNA (si-Pim), and from cells transfected with scrambled control siRNAs (si-control). Antibodies specific to β-actin were used as reference for equal loading.

To begin to address the molecular pathway through which PIM-2 exerts its G1 arrest and pro-apoptotic effects in HeLa cells, we utilized a protein array, containing 223 different antibodies (Panorama, Sigma), to compare proteins expressed in HeLa cells over-expressing PIM-2, versus proteins expressed in HA-control cells. Among proteins represented on the array, the CDK inhibitor, p57, showed an increase of about 70% in PIM-2 over-expressing cells. This increase was specifically confirmed at both the RNA and protein levels, by analyzing nuclear extracts from HA-PIM-2 expressing cells versus HA control cells, and the differences were just above the statistically significance of p<0.05 (Fig. 4A). We also performed a small scale phospho-site screen, using 18 phosphorylation site specific antibodies (Kinetworks™ custom protein screen), in which we detected a 50% increase in phosphorylation of T14/Y15 of CDK2 in HA-PIM-2 over-expressing cells. These results were verified by Western analysis of nuclear extracts from PIM-2 over-expressing cells and HA control cells, revealing that although CDK2 levels were equal in both samples, phosphorylation was significantly dominant in the HA-PIM-2 expressing cells (p<0.04, Fig. 4B). Notably, in Pim-2-silenced HeLa cells phosphorylation of CDK2 on T14/Y15 was significantly reduced (Fig. 4C), further supporting the effect of PIM-2 on CDK2 phosphorylation.

**Figure 5:**
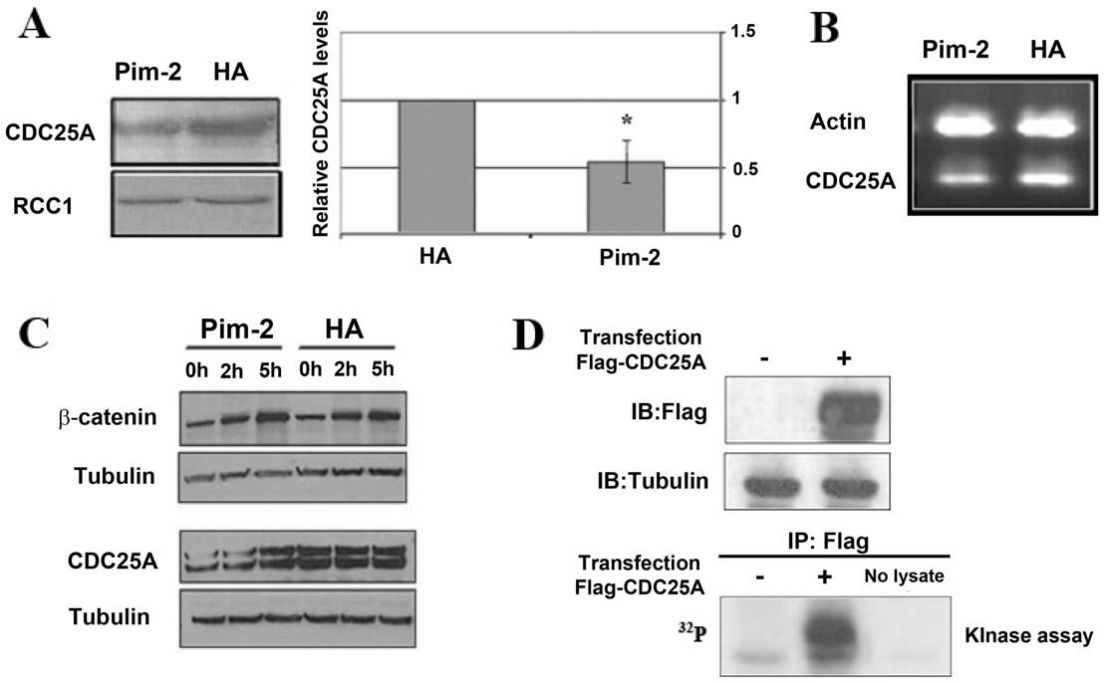
Down-regulation of CDC25A expression in HeLa cells over-expressing the 34 kDa form of PIM-2. (**A**) Western blot analysis of the CDC25A protein in nuclear extracts (40 µg) from cells over-expressing Pim-2 (Pim-2) and control cells transfected with empty HA vector (HA). Anti-RCC1 antibodies were used as reference for equal loading. The average relative level of CDC25A in Pim-2 over-expressing cells (Pim-2) compared to HA control cells (HA) is depicted in the right panel. Comparison was based on densitometric analysis of CDC25A signals normalized according to the RCC1 signal (average of three independent experiments). Average level of CDC25A in control cells was determined as 1. Asterisk represents statistically significant differences (p<0.05). (**B**) RT-PCR analysis of Cdc25A transcripts in Pim2 over-expressing cells (Pim-2) and in control cells (HA). Actin specific primers were used as reference for equal loading. (**C**) **PIM-2 promotes CDC25A degradation via the proteasome.** Western blot analyses of β-catenin (top panel), as control for proteasomal inhibition, and CDC25A (botom panel), were performed on total protein extracts (40 µg) from cells over-expressing Pim-2 (Pim-2) and control cells (HA) after treatment with the proteasome inhibitor, MG-132, for 0, 2 or 5 hours (h). Antibodies specific to tubulin were used as a reference for equal loading. (**D**) **PIM-2 directly phosphorylates CDC25A in an** ***in-vitro*** **kinase assay.** 293 cells were transfected (+), or not (−), with a Flag-tagged CDC25A expressing vector, and the tagged protein was immunoprecipitated using anti Flag antibodies. The immunoprecipitated protein was used as a substrate in a PIM-2 kinase assay (lower panel). Expression of the tagged protein was verified by Western analysis (upper panel), and equal loading was verified by stripping the blot and probing it with anti tubulin antibodies (middle panel).

CDC25A renders CDK2 active by dephosphorylating T14/Y15, and it can be regulated at the protein level, as part of the G1-S checkpoint, by phosphorylation dependent ubiquitin-mediated proteolysis [26]–[27]. We therefore, followed CDC25A levels after PIM-2 over-expression. We found that in HeLa cells over-expressing PIM-2, nuclear CDC25A levels were reduced by nearly 50% compared to control (p<0.05, Fig. 5A). RT-PCR analysis revealed a reduction (of about 20–30%) in the transcript level as well (Fig. 5B). To determine whether degradation of CDC25A at the proteasome also contributes to its down-regulation, HA PIM-2 expressing cells and HA control cells were cultured in the presence or absence of the proteasome inhibitor, MG-132, for 2 h or 5 h, and CDC25A levels were monitored. β-catenin, a protein known to be degraded at the proteosome, was used as a control. As can be seen in Figure 5C, β-catenin levels indeed increased over time after treatment with MG-132, independent of PIM-2 levels. Likewise, an elevation in CDC25A levels was observed in cells over-expressing PIM-2, in the presence of MG-132, whereas no such elevation could be detected in HA control cells. This suggested that PIM-2 over-expression, in our case, leads to increased degradation of CDC25A through the proteasome. Accordingly, silencing of Pim-2, via siRNA, led to increased CDC25A levels by about 40% (Fig. S7). Furthermore, in *in-vitro* kinase assays we demonstrated that PIM-2, both the commercial recombinant protein (Fig. 5D) and the immunoprecipitated HA-PIM, but not the HA-PIM-2KD (not shown), can directly phosphorylate CDC25A, raising the intriguing possibility that PIM-2-dependent phosphorylation of CDC25A might directly target it for proteasome mediated degradation. These results also suggest that reduced CDC25A levels contribute to G1 arrest through increased T14/Y15 phosphorylation on CDK2.

### Involvement of E2F-1 and p73 in PIM-2 mediated cell cycle arrest and apoptosis

**Figure 6:**
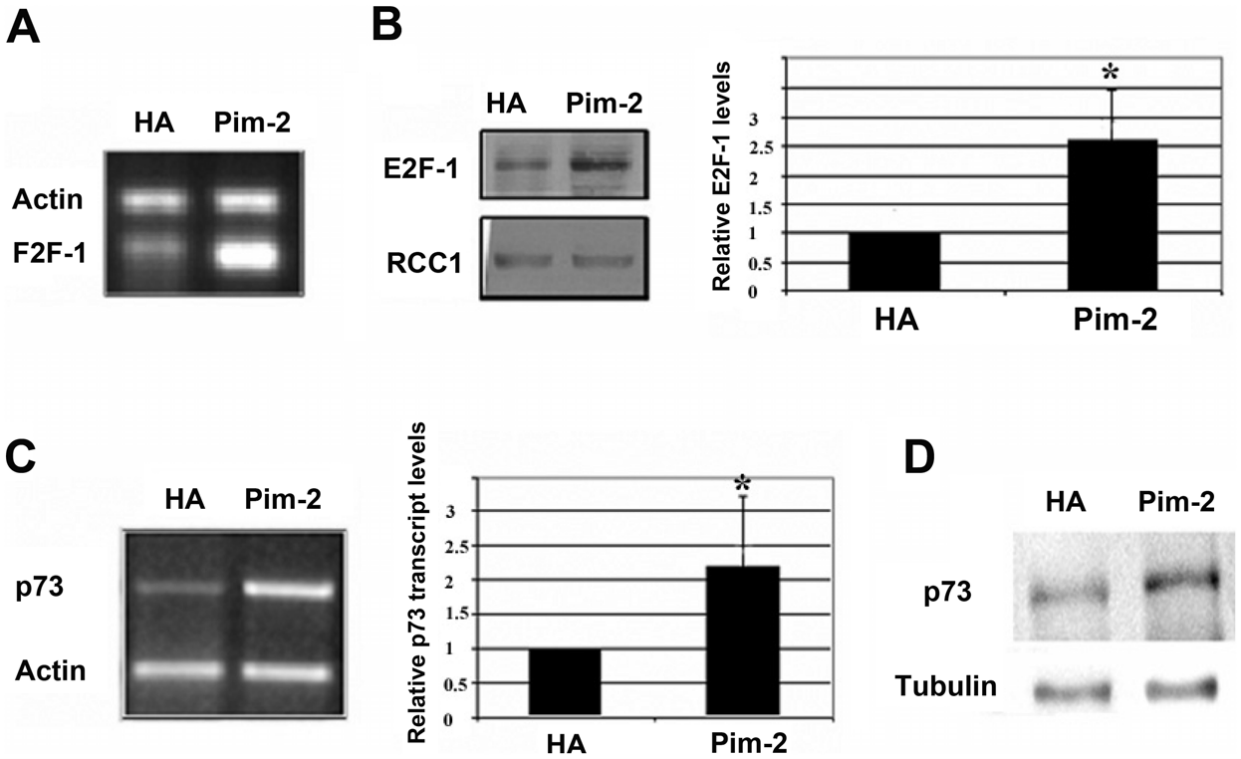
Up-regulation of E2F-1 and p73 expression in HeLa cells over-expressing the 34 kDa form of PIM-2. (**A**) RT-PCR analysis of E2F-1 transcripts in Pim2 over-expressing cells (Pim-2) and in control cells (HA). Actin specific primers were used as reference for equal loading. (**B**) Western blot analysis of the E2F-1 protein in nuclear extracts (40 µg) from cells over-expressing Pim-2 (Pim-2) and control cells with empty HA vector (HA). Blot was stripped and reprobed with antibodies specific to RCC1 as reference for equal loading. The average relative level of E2F-1 in Pim-2 over-expressing cells (Pim-2) compared to HA control cells (HA) is shown in the right panel. Comparison was based on densitometric analysis of E2F-1 signals normalized according to the RCC1 signal (average of three independent experiments). Average level of E2F-1 in control cells was determined as 1. Asterisk represents statistically significant differences (p<0.01). (**C**) RT-PCR analysis of p73 transcripts in Pim2 over-expressing cells (Pim-2) and in control cells (HA). Actin specific primers were used as a reference for equal loading. The average relative level of p73 in Pim-2 over-expressing cells (Pim-2) compared to HA control cells (HA) is shown in the right panel. Comparison was based on densitometric analysis of p73 signals normalized according to the β-actin signal (average of three independent experiments). Average level of p73 in control cells was determined as 1. Differences were statistically significant (p<0.01). (D) Western blot analysis of the p73 protein in total cell extracts (50 µg) from cells over-expressing Pim-2 (Pim-2) and control cells with empty HA vector (HA). Blot was stripped and reprobed with antibodies specific to β-tubulin as reference for equal loading.

**Figure 7:**
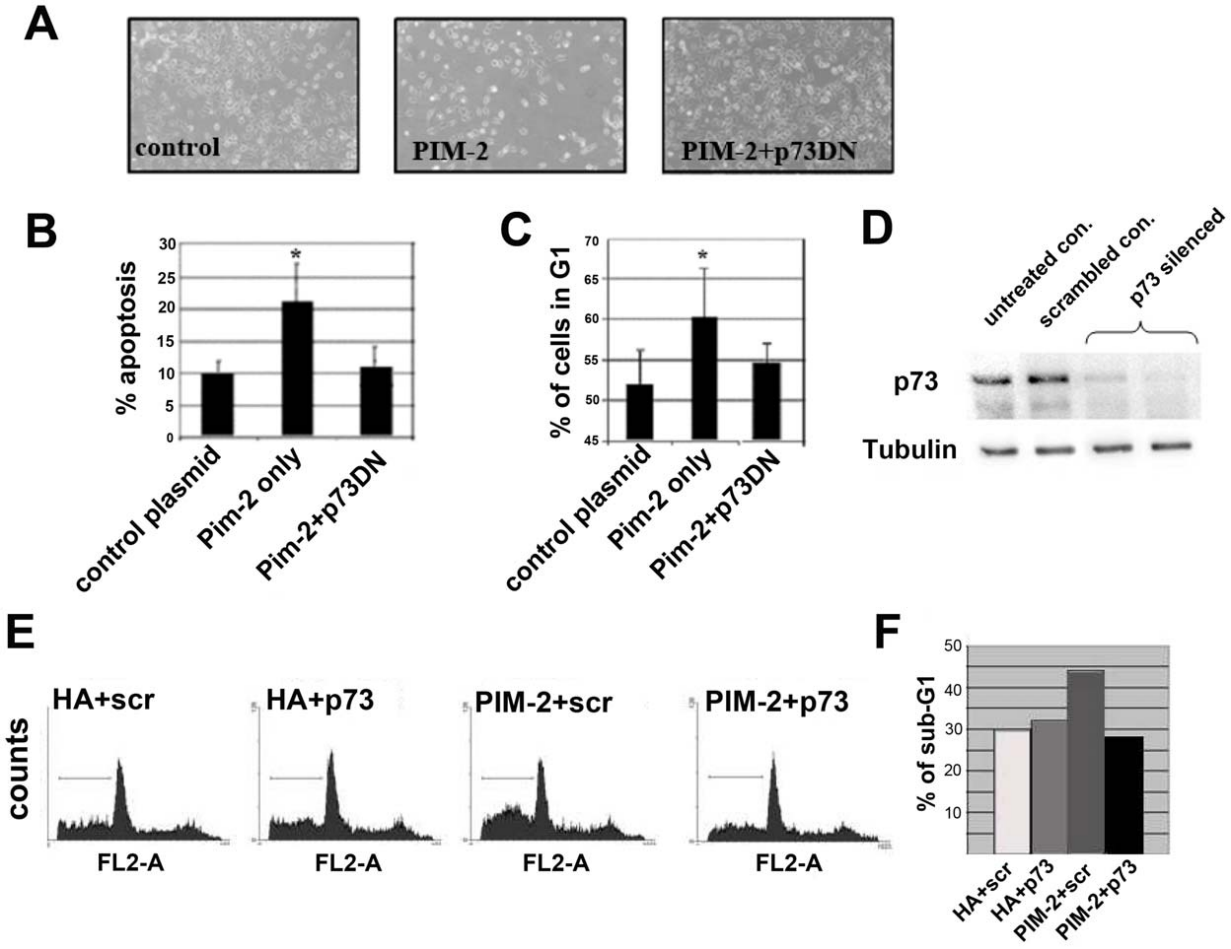
Dominant negative(DN) p73 or p73 silencing reverse the PIM-2 effect on cells over-expressing the 34 kDa PIM-2 isoform. (**A**) Light microscopic view (×40) of cells 48 hours after transfecting equal amounts of cells with an empty HA vector as control (control), HA-Pim2 plasmid alone (Pim-2) and a HA-Pim-2 plasmid together with a dominant negative form of p73 (Pim-2+p73DN), and under identical culture conditions. (**B**) Percent of sub-G1 apoptotic cells, and (**C**) percent of cells in the G1 phase of the cell cycle, in the specified cultures, as revealed by FACS analysis. Results represent an average of four independent experiments. Asterisk represents statistical significance p<0.01. (**D**) Western analysis showing siRNA-silencing of p73 compared to untreated control or control of cell treated with scrambled siRNA. (**E**) FACS analysis of cell cycle pattern of PI stained cells to which siRNAs were introduced [control scrambled (scr.) or p73-directed siRNA (p73)] 24 h prior to transfection with either empty HA plasmid (HA+scr. or HA+73) or HA-Pim-2 plasmid (PIM-2+scr. or PIM-2+73). FACS analysis was performed 76 h after transfection with plasmids. Horizontal line in each pattern indicates the channels that were included in calculation of the sub-G1 phase. (**F**) Percent of cells at the sub-G1 phase calculated from the cell cycle patterns presented in panel E.

Given the key role of the E2F-1 transcription factor in the G1 to S phase transition, we monitored changes in its level following PIM-2 over-expression. Surprisingly, we found that E2F-1 expression was consistently and significantly increased at both the RNA and protein levels following PIM-2 over-expression (p<0.01, Fig. 6A–B). These results seem to correspond to E2F-1's role as a pro-apoptotic factor (review in [28]). E2F-1 can induce apoptosis in a p53-dependent pathway [29]–[31], but it can initiate apoptosis in a p53-independent manner, by up-regulating the expression of the pro-apoptotic protein, p73 [32]–[33]. Since in HeLa cells, p53 is mostly inactivated by the HPV E6 ubiquitin ligase, we analyzed changes in p73 transcription following PIM-2 over-expression. Indeed, we found an apparent and statistically significant increase in p73 at both the transcript and the protein levels (p<0.01, Fig. 6C–D). To further assess the importance of p73 to the G1 arrest and apoptotic effects of PIM-2, we co-expressed PIM-2 with a dominant negative (DN) form of p73, p73DD [32], in HeLa cells, reasoning that if p73 indeed plays a role in the PIM-2 dependent G1 arrest and apoptosis, its DN form would rescue the cells from these effects. We found that indeed the p73 DN form has an apparent rescue effect on the G1 arrest and apoptotic phenomena. It decreased the percent of apoptotic cells to control levels and released the G1-arrest (Fig. 7A–C). To further verify that p73 indeed plays a key role in the PIM-2 activation of apoptosis, we used siRNA to silence p73 in HeLa cells prior to introducing HA-PIM-2 to these cells (Fig. 7D–E), and monitored the percentage of cells at the sub-G1/apoptotic phase compared to scrambled siRNA control. We found that while scrambled siRNA did not affect the PIM-2-dependent elevation in apoptosis, silencing of p73 prior to HA-PIM-2 expression blocked this effect with sub-G1 levels as in HA controls (Fig. 7E–F). We, therefore, concluded that PIM-2 can activate cell cycle arrest and apoptosis in HeLa cells in a p73-dependent manner.

## Discussion

Previously identified substrates of the PIM-2 kinase, share an oncogenic promoting function as either anti-apoptotic/survival factors, transcription factors that can increase expression of oncogenic proteins, or cell cycle regulators (review in [34]). Moreover, increased expression of PIM-2 was directly associated with various malignancies, such as non-Hodgkin's lymphoma, CLL and prostate cancer [10]–[13], further supporting the oncogenic function ascribed to PIM-2, and justifying targeting its kinase activity as a beneficial therapeutic approach [21], [35]. In this study, however, we found that under certain circumstances PIM-2 might exert cell cycle arrest and pro-apoptotic effects, suggesting that PIM-2 may play a dual role and that targeting its activity might not always be therapeutically beneficial. Interestingly, PIM-1 expression has also been correlated with poor prognosis in haematopoietic malignancies and with good prognosis in other malignancies (review in [34]). It is possible that the differential function of PIM-2 is cell type dependent. However, given that similar results were obtained in more than one cell line, this phenomenon seems to be of a more general nature. Our finding that only the 34 kDa isoform, but not the 41 kDa isoform, could activate the cell cycle arrest and pro-apoptotic effects, suggest that the balance between these two isoformes might affects PIM-2's overall effect. Moreover, the fact that the pro-apoptotic effect of the 34 kDa isoform was even intensified in a background of silencing the endogenous PIM-2 (Fig. S6) might suggest that the 41 kDa has a somewhat moderating effect on the activity of the 34 kDa isoform. This can be the outcome of both isoformes sharing the same substrates but with differential phosphorylation efficiencies, as has been reported in the mouse PIM-2 where the 41 kDa isoform is less active than the 34 kDa isoform [15]. An additional potential regulatory level that can be considered is that the balance between nuclear and cytoplasmic PIM-2 (mainly the 34 kDa isoform) is important to determine PIM-2's overall effect. This latter hypothesis is consistent with the results of Dai et al. [13], reporting a shift from a predominant nuclear expression of PIM-2 in normal prostate epithelium cells, to an increased cytoplasmic expression in prostate cancer cells (PCa). These authors further reported that increased nuclear expression of PIM-2 in perineural invasion (a common pathological phenomenon proposed to be the dominant pathway through which PCa spreads beyond the prostate) was associated with decreased proliferation. The experimental results reported herein, undoubtedly disrupted the cytoplasmic/nuclear balance, exposing nuclear substrates to increased PIM-2-dependent phosphorylation. This concept of a protein exhibiting rather opposing effects depending on its sub-cellular localization has been reported in other systems as well. One such example is the cyclin A-CDK2 complex in mouse mesangial cells, where in its nuclear form it functions to promote cell division, whereas following an apoptotic stimulus, it accumulates in the cytoplasm where it actively functions to promote apoptosis [36]–[37]. Whatever the reasons for the differential effects of PIM-2 are, this study suggests that while targeting PIM-2's kinase activity might indeed be beneficial chemotherapeutically in certain malignancies, it might be devastating in other cancers in which activating PIM-2 might actually reduce the malignant phenotype. This of course necessitates in depth study of the molecular pathways through which each effect is exerted.

Up-regulation of p57 and increased T14/Y15 phosphorylation of CDK2 can explain, at least in part, the G1 arrest in the PIM-2 expressing cells. The p57 protein, as a member of the Cip/Kip family of CDK2 inhibitors, binds to and inhibits cdk2/cyclin E/cyclin A complexes during the G1 phase [38]–[39]. G1 arrest due to increased levels of p57 have been demonstrated in several cell systems including primary human hematopoietic cells [40], and HeLa cells following treatment with the synthetic glucocorticoid, dexamethasone [41]. Moreover, increased levels of p57 were shown to sensitize HeLa cells to apoptosis after treatment with cytotoxic drugs, such as staurosporine and etoposide [42], and to specifically promote the mitochondrial apoptotic pathway in cancer cells, via a mechanism that does not require p57-mediated inhibition of CDK [43]. As to the T14/Y15 phosphorylation, it is well known that phosphorylation at this site negatively regulates the catalytic activity of CDK2, and that CDK2 must undergo dephosphorylation at this site by CDC25A phosphatase prior to the G1 to S-phase transition. The reduced CDC25A levels, we found in nuclear extracts of PIM-2 over-expressing cells, seem to account, at least in part, for the increased T14/Y15 phosphorylation of CDK2, thus contributing to the G1 arrest.

Maintaining CDC25A balanced level during the various cell cycle stages requires its ubiquitylation by the SCF^βTrCP^ ubiquitin ligase complex and proteasomal degradation. Targeting CDC25A for SCF^βTrCP^ ubiquitylation depends on its phosphorylation on various Ser residues, mainly by the Chk1 and p38 kinases [27], [44]. Moreover, following DNA damage, CDC25A is rapidly phosphorylated by Chk1 and Chk2, and possibly by other kinases, targeting it for ubiquitylation and degradation, thus imposing G1 arrest and DNA synthesis block [27], [45]. The proteasome-dependent degradation of CDC25A, seen in this study upon PIM-2 over-expression, suggests that PIM-2 promotes CDC25A phosphorylation that triggers its ubiquitylation. The fact that PIM-2 can directly phosphorylate CDC25A, stresses the possibility that PIM-2 can directly target CDC25A for ubiquitylation and degradation, although this should be further validated. Interestingly, PIM-1 was also reported to phosphorylate CDC25A, although this phosphorylation stabilizes CDC25A and increases its phosphatase activity [46].

The reduction in Cdc25A transcript level, shown in this study, is puzzling in light of the fact that E2F-1, a transcriptional activator of Cdc25A (together with c-Myc), was up-regulated following PIM-2 expression. Moreover, c-Myc itself has been shown to be stabilized by PIM-2 in a way that enhances its transcriptional activity [19]. One possible explanation relates to various co-factors that can interact with E2F-1 or c-Myc, turning them into effective transcriptional repressors. For example, the Cip/Kip p21 protein, known for its CDK inhibitory function, was shown to interact with the E2F-1 and STAT3 transcription factors and to suppress their trans-activating activity [47]–[48]. Moreover, p21 was shown to specifically interact with E2F-1 and STAT3 at the Cdc25A promoter following DNA damage and to suppress its transcription [49]. The possibility that one of the Cip/Kip proteins, or some other cofactors, indeed interfere with Cdc25A transcription in HeLa cells upon PIM-2 over-expression, is currently under investigation.

Our results indicated up-regulation of E2F-1 following PIM-2 over-expression, possibly by stabilization and activation of its transcriptional activator c-Myc [19], suggesting that it functions as a pro apoptotic factor in this system [28]. E2F-1 can induce apoptosis in a p53-dependent pathway [29]–[31] or through a p53-independent pathway by up-regulating p73 or Apaf-1 [32]–[33], [50]. We found that p73 expression was indeed up-regulated upon over-expression of the 34 kDa isoform of PIM-2. Moreover, co-expression of a dominant negative form of p73, that binds to p73 and prevents it from activating p53-responsive promoters (32), with the HA-PIM-2 34 kDa isoform, as well as siRNA-mediated silencing of p73 in these cells, abrogated the pro-apoptotic and G1 arrest effects of PIM-2, identifying p73 as a major mediator of these effects in our system. p73 can induce cell death via different pathways. It can do so by activating the ER stress pathway, it can activate the mitochondrial pathway by directly activating transcription of PUMA and mitochondrial translocation of Bax, and there is evidence that it can activate expression of the CD95 death receptor [51]–[52]. Nevertheless, Blint et al. [53] reported that p73β, an isoform of p73, can activate expression of the p57 CDK inhibitor. Later it was shown that p73β-mediated apoptosis requires p57 [54]. This suggests a possible involvement of p57 in the p73-dependent pro-apoptotic and G1 arrest effects in the PIM-2 over-expressing cells.

The Akt and the Pim kinase families, both regarded as survival kinases, display close, although somewhat complicated, interrelationship. On one hand, both kinase families share many common substrates [23], and on the other hand it has been shown that over-expression of a Pim-1 dominant negative form in cardiomyocytes, increases Akt and phospho-Akt (Ser473) levels, demonstrating reciprocal feedback signaling between Akt and Pim-1 [25]. These authors further suggest that Pim-1 might be a downstream effector of Akt since inactivation of Pim-1 induced apoptosis in cardiomyocytes, effect that could not be reversed by over-expression of Akt. Reciprocal relationship between Pim and Akt was reported also in v-Abl-transformed pre-B cells, where Akt and phospho-Akt levels were increased in transformed cells from triple Pim knockout mice [55]. Furthermore, these authors showed that transformed cells from triple Pim knockout mice were less sensitive to imatinib treatment when they express a constitutive active mutant Akt, suggesting that Akt can promote survival of Pim-deficient cells. Given this Akt-Pim relationship, an intriguing question, which has not been addressed in this work, is what role dose Akt play in our Pim-2-dependent cell growth retardation. Does Akt expression and/or activity are down-regulated due to Pim-2 over-expression? This issue must await further investigation.

**Figure 8:**
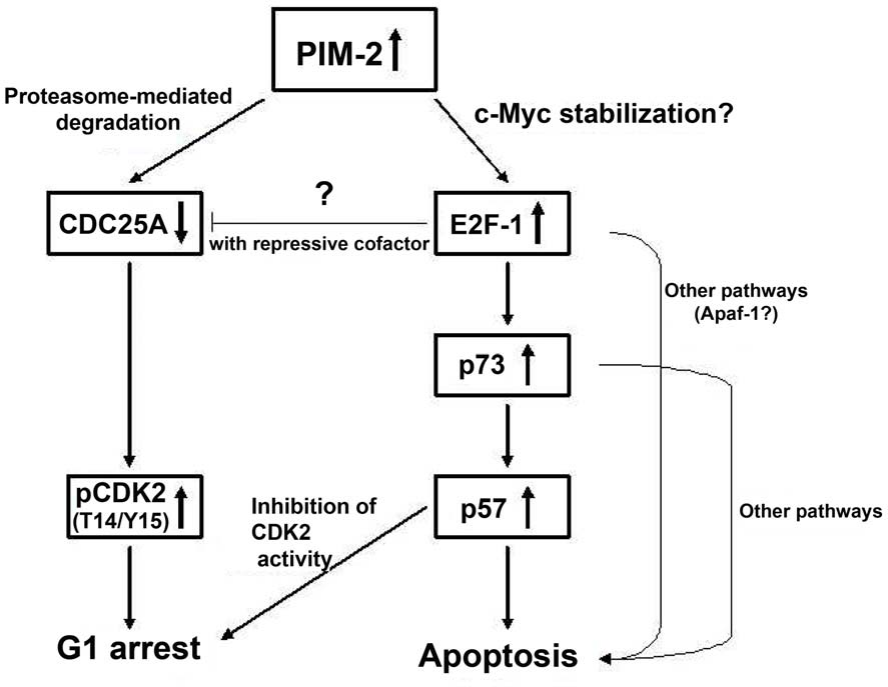
A proposed model explaining the molecular pathway through which PIM-2 exerts G1-arrest and promotes apoptosis.

In conclusion, we suggest that PIM-2 over-expression, that disrupts either the balance between the 34 kDa and the 41 kDa isoformes or the cytoplasmic/nuclear balance of PIM-2, (or both) leads to cell cycle arrest and increased apoptosis. We propose a model of transcriptional cascade in which up-regulation of E2F-1, possibly by c-Myc stabilization, results in p73 up-regulation, which in turn activates expression of various apoptotic factors, including p57, which is required for p73-mediated apoptosis. Increased p57 levels also contribute to G1 arrest by inhibiting CDK2 (Fig. 8). In addition, our results suggest that PIM-2 activates proteasome-mediated degradation of CDC25A by targeting it (directly or indirectly) to ubiquitylation (Fig. 8). Reduced CDC25A levels increase T14/Y15 phosphorylation of CDK2 and hence promote G1 arrest (Fig. 8).

## Materials and Methods

### Cell culture and transfection

HeLa – human epitheloid cervix carcinoma, U2OS – human osteosarcoma, and B16 – mouse melanoma, cells were obtained from the American Type Culture Collection (ATCC). EHEB – human B-cell chronic leukemia cells were obtained from the DSMZ Human and Animal Cell Lines collection. Other cell, including: PC-3 – human prostate adenocarcinoma [56], HCT 116 - human colorectal carcinoma [57], HT-29 – human colon adenocarcinpma [58], HEK-293 – human embryonic kidney [59], MCF7 – human breast adenocarcinoma [60], K-562 – human chronic myelogenous leukemia [61], HL-60 – promyelocytic leukemia [61], Raji – human Burkitt's lymphoma lymphoblasts [62], and NALM6 – human pre-B leukemia [63], were obtained from neighboring laboratories. All cells were cultured in either DMEM or RPMI 1640 medium, supplemented with 10% fetal bovine serum, L-glutamine (2 mM) and 1%Penicillin-Streptomycin-Nystatin (Gibco). Cells were maintained at 37°C with 5% CO_2_ in a humidified chamber. Transfections of equal amounts of cells (4×10^5^ cells/10 cm plate) were carried out using JetPEI transfection reagent (Biomol, Germany) according to the manufacturer's instructions. For p73 silencing, p73-directed siRNAs (Sigma) were introduced to HeLa cells using the PepMute™ siRNA transfection reagent (Signagen laboratories, Ijamaville MD) according to the manufacturer protocol. 24 hours (h) later cell were transfected with the HA-Pim-2 construct as mentioned above. After additional 24 h the culture medium was replaced and a second siRNA transfection reaction was performed with half of the amount of siRNA, and 48 h later cells were harvested and analyzed. For Pim-2 silencing, Pim-2-directed siRNAs (Ambion or Sigma-mix) were introduced to HeLa cells as detailed above for p73.

### Plasmids

The HA-Pim-2 vector was created by subcloning a PCR fragment encoding the entire 34 kDa isoform of human Pim-2 into the mammalian expression vector pCMV-HA (Clontech) at the *EcoRI* and *NotI* sites (forward primer- 5′ccccgaattcccatgttgaccaagcctcta3′; reverse primer- 5′ccccgcggccgccaacaaatgtccatctatccc3′). The Pim-2-Flag vector was created by subcloning a PCR fragment encoding the entire 41 kDa isoform of human Pim-2 into the mammalian expression vector p3XFLAG-CMV-14 (Sigma) at the *EcoRI* and *BamHI* sites (forward primer- 5′cggaattctgctttccaccctggcgccc3′; reverse primer- 5′cgggatccgggtagcaaggaccaggccaaag3′). A kinase dead form, HA-Pim-2KD was constructed as described [14]. For Myc-His-tagging, a PCR fragment encoding either sequence was subcloned into the pCDNA3.1/Myc-His vector (Invitrogen) at the *NotI* and *XbaI* sites (forward primer- 5′gcatagcggccgcgttgttatgtacccatacgatgtt3′; reverse primer- 5′gcatatctagagggtagcaaggaccaggc3′). A p73 DN vector (p73DD, [32]) was a gift from Prof. Doron Ginsberg (Bar-Ilan University, Israel).

### RT-PCR analysis

RNA was isolated using EZ-RNA II kit (Biological Industries, Israel). 1 µg of total RNA was used for cDNA preparation, using the RevertAid M-MuLV Reverse Transcriptase kit (Fermentas), and 5 µl of the cDNA product was used for PCR amplification, using the REDTaq polymerase (Sigma). The following primers were used for monitoring expression of the various genes: p73-(forward- 5′ccagctccaccttcgacacc3′; reverse- 5′ccggtagtggtcctcatcag3′), CDC25A-(forward- 5′accgtcactatggaccagc3′; reverse- 5′ttcagagctggactacatcc3′), E2F-1-(forward- 5′tgacctgctgctcttcg3′; reverse- 5′gttcaggtcgacgacac3′), p57-(forward- 5′cggcgatcaagaagctgtcc3′; reverse- 5′cccagcgcccttccaac3′), actin-(forward- 5′cctgaccctgaagtacccc3′; reverse- 5′ggtagtcagtgaggtcgcg3′).

### Protein extraction and analysis

Nuclear and cytoplasmic extracts were prepared according to Gil et al. [64]. Protein concentration assays were performed using the Bradford Reagent (Bio-Rad). Western blotting was performed according to standard procedure. Signal was visualized by chemiluminescence detection reagents (Pierce). Antibodies used as primary antibodies included: Rabbit anti-PIM-2 [10], Mouse anti-HA (Covance, Berkeley), Rabbit anti-FLAG (Immunology Consultants Laboratory), Goat anti-RCC1 (Santa Cruz Biotechnology), Mouse anti-p57^Kip2^ (Sigma), Rabbit anti-CDK [pTpY^14/15^] (Biosource), Rabbit anti-CDK2 (Santa Cruz Biotechnology), Rabbit anti-E2F-1 (Santa Cruz Biotechnology), Rabbit anti-CDC25A (Santa Cruz Biotechnology), Rabbit mAb to p73 (abcam), Mouse anti-β-Actin (Oncogene Research Products), and Mouse anti-β-Tubulin (Developmental Studies Hybridoma Bank). Secondary antibodies included: Mouse anti-Rabbit IgG HRP-conjugated (Sigma); Goat anti-Mouse IgG HRP-conjugated (Bio-Rad); and Donkey anti-Goat IgG HRP-conjugated (Jackson Laboratories).

For immunoprecipitation (IP), 25–30 µl of proteinA/G agarose beads (Santa cruz Biotechnology) were incubated with 1 µl of the relevant antibody in 150 µl IP buffer (20 mM Tris pH = 7.5, 150 mM NaCl, 1 mM EDTA, 1 mM EGTA, 0.5%NP-40, 2.5 mM Sodium pyrophosphate, 1 mM β-glycerophosphate, 1 mM Na_3_VO_4_, 1 mM PMSF, Protease inhibitor cocktail) for two hours at 4°C. The beads were washed twice with IP buffer and then incubated over-night at 4°C with the protein lysate (containing 500–1000 µg protein), which was pre-treated with 20 µl A/G agarose beads to exclude proteins that bind the beads non-specifically. Beads were then washed (×5) with IP buffer (for Western analysis) or with kinase assay buffer (60 mM Hepes (PH = 7.5), 3 mM MgCl_2_, 3 mM MnCl_2_, 3 mM Na_3_VO_4_, 1.2 mM DTT), and finally resuspended in 30–50 µl of the corresponding buffer.

### *In-vitro* kinase assay

Either recombinant PIM-2 (Abgen), or immunoprecipitated HA-PIM-2 (or HA-PIM-2KD), were assayed *in-vitro*, using recombinant BAD (Santa cruz Biotechnology) as a substrate. 1 µg BAD was added to 47 µl of immunoprecipitated HA-PIM-2-beads or to 0.1 µg of recombinant PIM-2 in 47 µl kinase assay buffer, together with 3 µl of a γ^32^P-ATP solution [3 µl of γ^32^P-ATP (250 µCi)+3 µl of cold ATP (1 mM)+24 µl H_2_O]. The reaction was incubated 1 hour at room temperature (gently resuspending the beads every 10 minutes). Following incubation, samples were boiled for 5 minutes in sample buffer and separated in 10% SDS-PAGE. The Gels were then dried in a gel-drier and exposed over-night to an X-ray film. For PIM-2/CDC25A kinase assay, 0.1 µg of recombinant PIM-2 (or precipitated HA-PIM-2 beads) was added to immunoprecipitated CDC25A-Flag-beads in 47 µl kinase assay buffer, and the reaction was carried out as described above.

### MassSpectrometry analysis

293T cells were transiently transfected with the HA-Pim-2 vector and seeded on 10 culture plates (10 cm). 48 hours later, total protein extracts were pooled, and HA-PIM-2 was immunoprecipitated using anti-PIM-2 antibodies. The precipitated proteins were resolved on 10% PAGE and stained with Coomasie Brilliant Blue (sigma). The two PIM-2 bands were cut from the gel and sent to MassSpectrometry analysis in the Israeli national proteomic center at the Technion (Hifa, Israel).

### Phosphatase and glycosidase assay

The Lambda protein phosphatase Kit (New England BioLab) was used for the phosphatase assay, for either cytoplasmic or nuclear protein extracts, according to manufacturer protocol. For the glycosidase assay, 40 µg of either cytoplasmic or nuclear protein extracts were boiled in 1%SDS for 5 minutes and then diluted 1∶10 in glycosidase buffer (EDTA 0.02 M, Triton X-100 0.55% and β-mercaptoethanol 1% all in PBS). 1 µl of recombinant N-Glycosidase F enzyme (Roche) was added, and the reaction was incubated over-night at 37°C. Following incubation, samples were boiled in sample buffer and analyzed by Western blotting.

### MTT assay

For cell viability assessment using the MTT assay, U2OS Tet-on cells were plated in 96 wells plate (2×10^3^ cells/well) and 24 h later doxycycline, at different concentrations, was added to the cultures. After additional 96 h the doxycycline-containing medium was replaced by 1 mg/ml MTT solution in PBS (pH-7.4) for 2 h at 37°C. The MTT solution was then removed and DMSO was added to dissolve the insoluble formazan product. The absorbance of the colored solution was read by a spectrophotometer at 550 nm.

## Supporting Information

Figure S1 **Immunocytochemical analysis of PIM-2 distribution in various cell lines.** Rabbit anti-PIM-2 antibodies and Alexa 488 conjugated anti-rabbit secondary antibodies (green) were used for staining. Nuclei were stained with propidium iodide (PI-red). Control cells were stained with pre-immune serum and secondary antibodies. Bar represents 15 µm.
(TIF)

Figure S2 **(A) Phosphatase assay and (B) Glycosidase assay to cytoplasmic (C) and nuclear (N) PIM-2.** Nuclear or cytoplasmic proteins (50 µg), from the indicated cell lines, were treated (+) or not (−) with Lambda protein phosphatase or with recombinant N-Glycosidase F enzymes, respectively. Following the enzymatic treatment proteins were analyzed by Western blotting using anti PIM-2 antibodies as primary antibody and HRP conjugated anti rabbit IgG as secondary antibody. The membranes were stripped twice and reacted once with anti RCC1 antibody as control for nuclear proteins, and once with anti tubulin antibody as control for cytoplasmic proteins. Dcpia and MSP were used as controls for the efficiency of the phosphatase and glycosidase assays, respectively.
(TIF)

Figure S3 **Pim-2 silencing in HeLa cells using Pim-2-directed siRNAs (Ambion).** (A) Western blot of proteins extracts from Pim-2 silenced cells (si-Pim-2) and from control cells transfected with scrambled control siRNAs (si-control). (B) Light microscope images (×40) of cells 48 hours after transfecting equal amounts of cells with either Pim-2-derected siRNAs (si-Pim2) or scrambled control siRNA (si-contro), and under identical culture conditions. Right panel - FACS analysis of cell cycle distribution of PI stained cells 48 hours after transfection with either −2-derected siRNAs (si-Pim2) or scrambled control siRNA (si-contro).
(TIF)

Figure S4 **Kinase assay to: (A) immunoprecipitated HA-PIM-2 and HA-PIM-2KD proteins, and (B) commercial recombinant PIM-2, as a positive control, using recombinant BAD as a substrate.** HA-PIM was immunoprecipitated using the anti HA antibody (IP:HA). Western analysis of the HA-immunoprecipitated protein, as well as of the total protein lysate (input), are depicted (IB:HA).
(TIF)

Figure S5 **Doxycycline dose-dependent expression of either HA-PIM-2 or HA-PIM-2KD in a stable Tet-on-inducible system in U2OS cells.** (A) Total protein extracts from cultures treated with the indicated concentrations of doxycyclin, were analyzed by Western blotting using anti-HA and anti-PIM-2 antibodies for detection of the recombinant proteins (IB:HA and IB:Pim, respectively). (B) Survival of U2OS Tet-on cells expressing either HA-PIM-2 (U2OS Tet-on Pim-2), or HA-PIM-2-Kinase Dead (U2OS Tet-on Pim-2KD), 96 h after activation of expression by increasing concentrations of Doxycycline, as indicated. Survival rates were determined by the MTT assay, compared to cells not treated with Doxycycline. Tet-on U2OS cells with no Pim-2 constructs were used as control (U2OS Tet-on). This panel represents an experiment that was executed in quadruplicates with very small standard error values. (C) Percent cells at the different phases of the cell cycle, as determined by FACS analysis. All cells (as indicated in panel B) were exposed to Doxycycline (2 µg/ml) for 96 h.
(TIF)

Figure S6 **Differential effects of over-expressing either the 34 kDa or 41 kDa isoformes in endogenous PIM-2 silenced cells.** (A) Western blots showing silencing of endogenous PIM-2 in U2OS cells, using anti-PIM-2 antibodies (upper panel), over-expression of HA-tagged 34 kDa isoform using anti-HA antibodies (middle panel), and over-expression of Flag-tagged 41 kDa isoform using anti-Flag antibodies (lower panel). Blots were stripped and reprobed with anti-tubulin antibodies for equal loading assessment. (B) Sub-G1 analysis of U2OS cells treated with either PIM-2 shRNA or non-specific (NS) shRNA as control, each transfected with either the HA-tagged 34 kDa encoding plasmid, the Flag-tagged 41 kDa encoding plasmid, or with an empty HA vector as control. Percent of cells at the sub-G1 phase is indicated in each panel. (C) Average percentage of cells (treated as described in panel B) in sub-G1 phase. Asterisks represent statistically significant differences (p<0.05).
(TIF)

Figure S7 **Analysis of CDC25A levels in PIM-2 silenced cells.** CDC25A levels were increased by about 40% in PIM-2 silenced cells in two independent experiments using two different sets of siRNS oligos to silent PIM-2 (Ambion – si-Pim-2 or Sigma – si-Pim-2 mix). Scrambled si-RNAs were used for control experiments. Tubulin antibody was used as a control for equal protein loading and served as reference for densitometric analysis.
(TIF)
