# Supplementary figures and images for: Activation of Cell Cycle Arrest and Apoptosis by the Proto-Oncogene Pim-2
Source: PLoS One. 2012 Apr 10;7(4):e34736. doi: 10.1371/journal.pone.0034736 (PMC3323563; doi:10.1371/journal.pone.0034736)

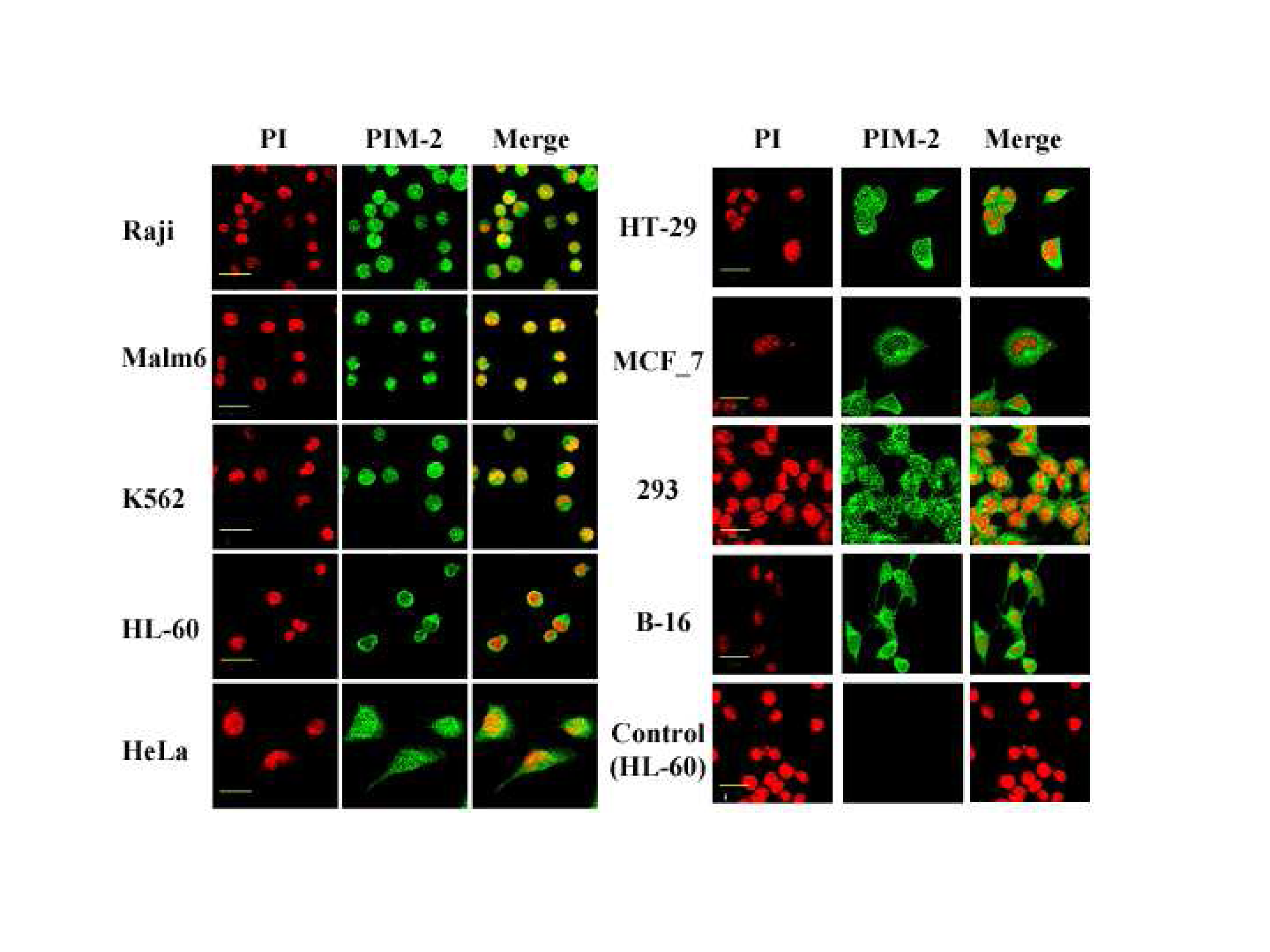

Supplement: Figure S1 — Immunocytochemical analysis of PIM-2 distribution in various cell lines. Rabbit anti-PIM-2 antibodies and Alexa 488 conjugated anti-rabbit secondary antibodies (green) were used for staining. Nuclei were stained with propidium iodide (PI-red). Control cells were stained with pre-immune serum and secondary antibodies. Bar represents 15 µm. (TIF) [file pone.0034736.s001.tif]

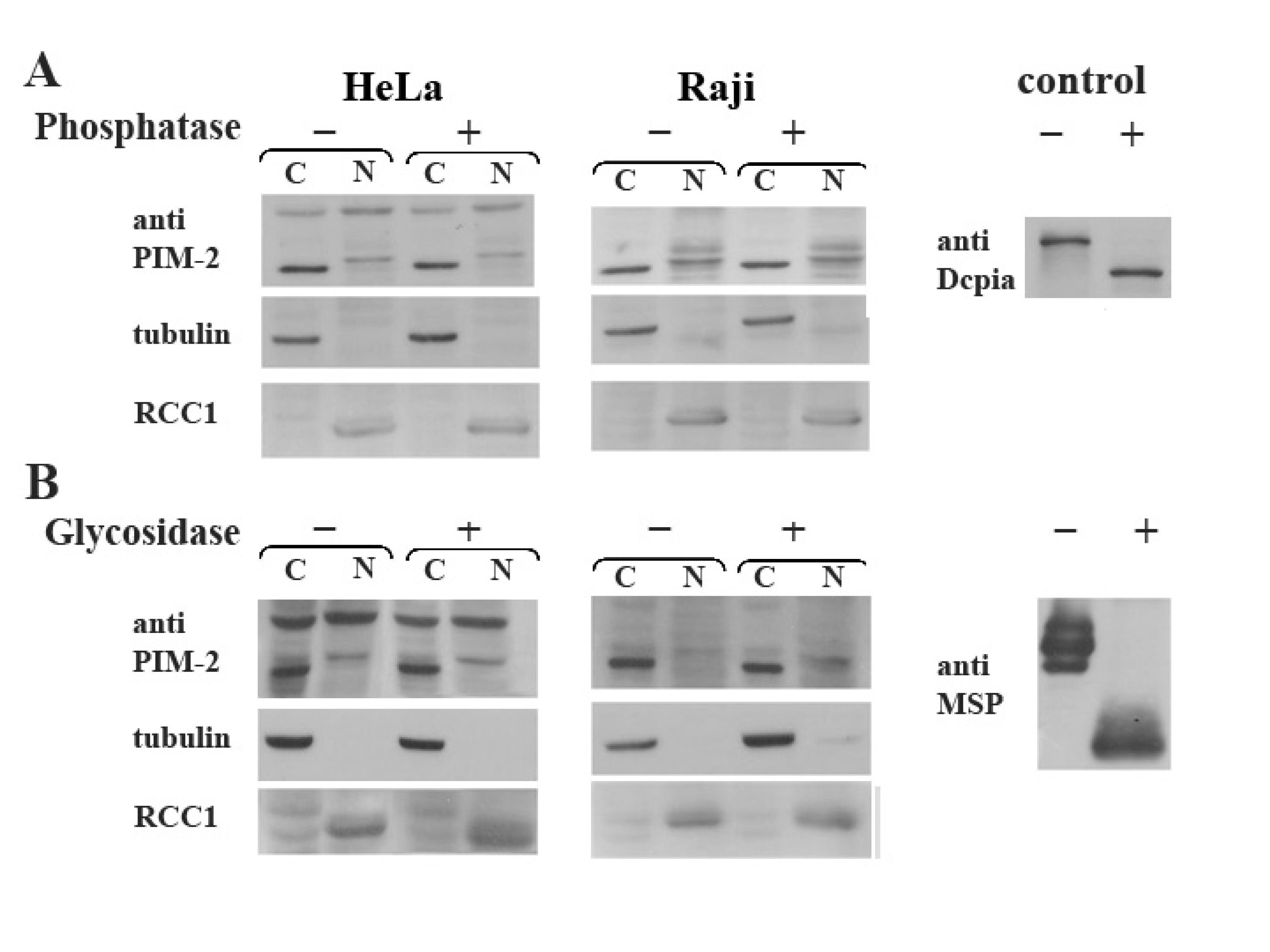

Supplement: Figure S2 — (A) Phosphatase assay and (B) Glycosidase assay to cytoplasmic (C) and nuclear (N) PIM-2. Nuclear or cytoplasmic proteins (50 µg), from the indicated cell lines, were treated (+) or not (−) with Lambda protein phosphatase or with recombinant N-Glycosidase F enzymes, respectively. Following the enzymatic treatment proteins were analyzed by Western blotting using anti PIM-2 antibodies as primary antibody and HRP conjugated anti rabbit IgG as secondary antibody. The membranes were stripped twice and reacted once with anti RCC1 antibody as control for nuclear proteins, and once with anti tubulin antibody as control for cytoplasmic proteins. Dcpia and MSP were used as controls for the efficiency of the phosphatase and glycosidase assays, respectively. (TIF) [file pone.0034736.s002.tif]

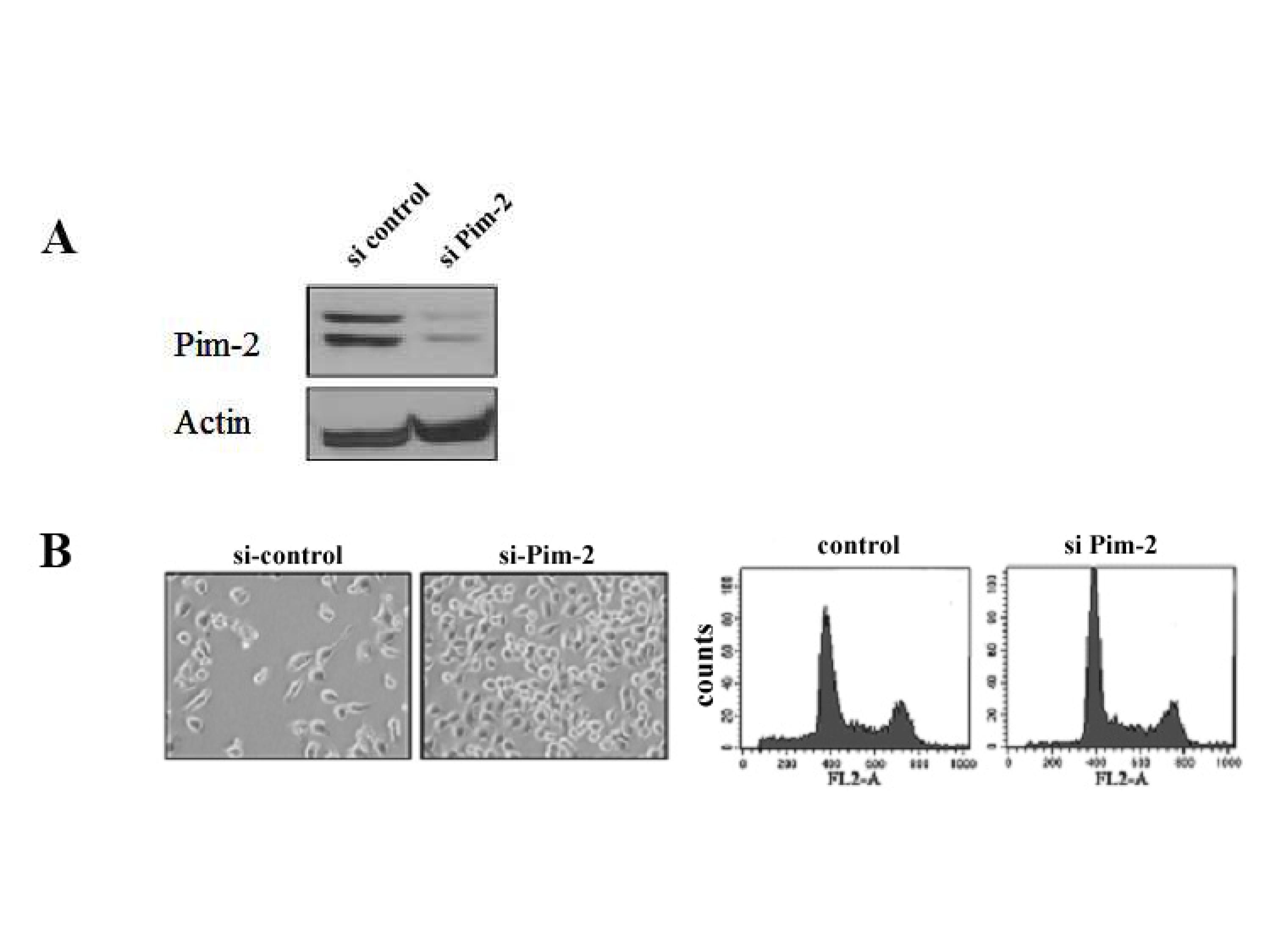

Supplement: Figure S3 — Pim-2 silencing in HeLa cells using Pim-2-directed siRNAs (Ambion). (A) Western blot of proteins extracts from Pim-2 silenced cells (si-Pim-2) and from control cells transfected with scrambled control siRNAs (si-control). (B) Light microscope images (×40) of cells 48 hours after transfecting equal amounts of cells with either Pim-2-derected siRNAs (si-Pim2) or scrambled control siRNA (si-contro), and under identical culture conditions. Right panel - FACS analysis of cell cycle distribution of PI stained cells 48 hours after transfection with either −2-derected siRNAs (si-Pim2) or scrambled control siRNA (si-contro). (TIF) [file pone.0034736.s003.tif]

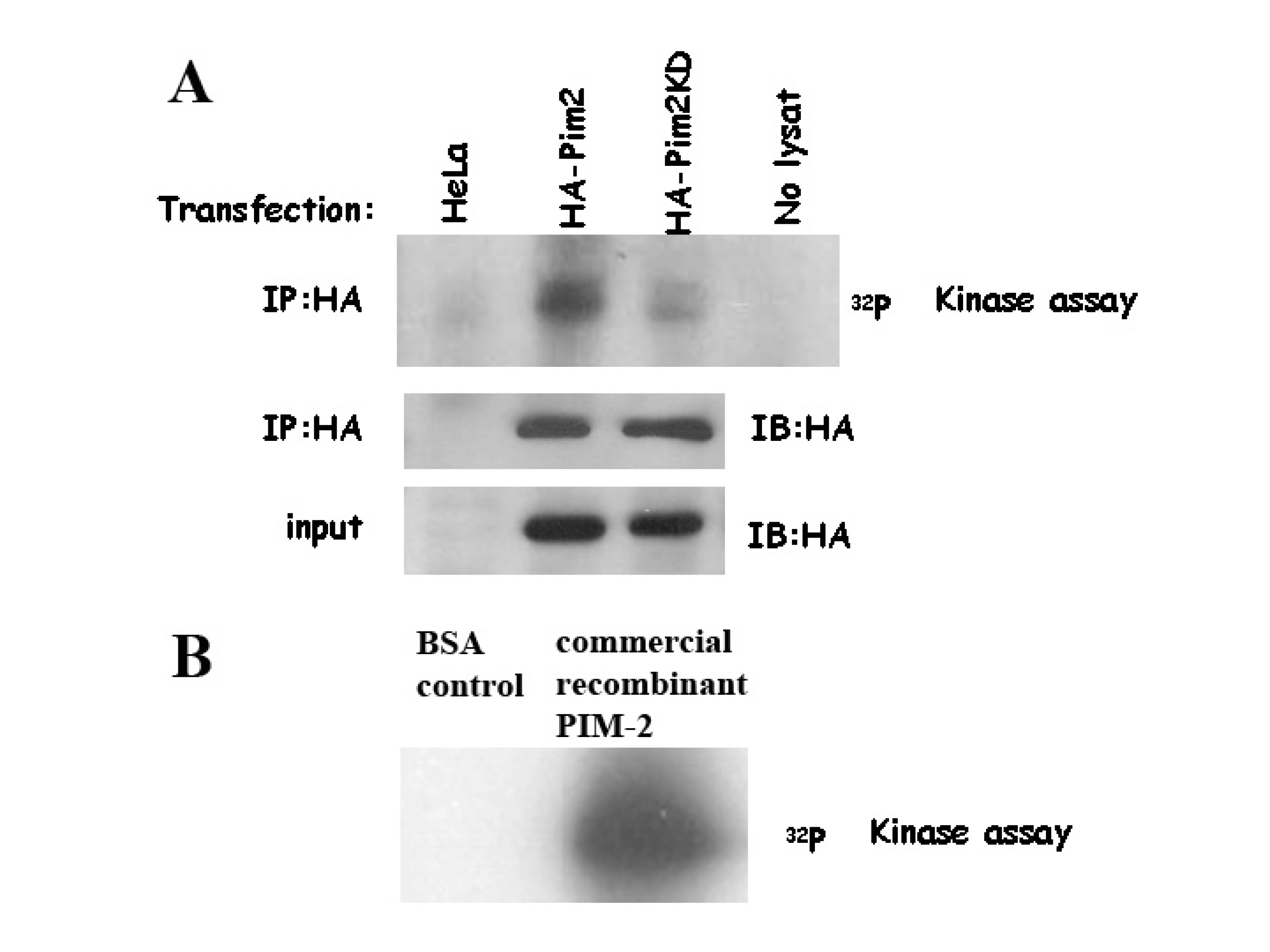

Supplement: Figure S4 — Kinase assay to: (A) immunoprecipitated HA-PIM-2 and HA-PIM-2KD proteins, and (B) commercial recombinant PIM-2, as a positive control, using recombinant BAD as a substrate. HA-PIM was immunoprecipitated using the anti HA antibody (IP:HA). Western analysis of the HA-immunoprecipitated protein, as well as of the total protein lysate (input), are depicted (IB:HA). (TIF) [file pone.0034736.s004.tif]

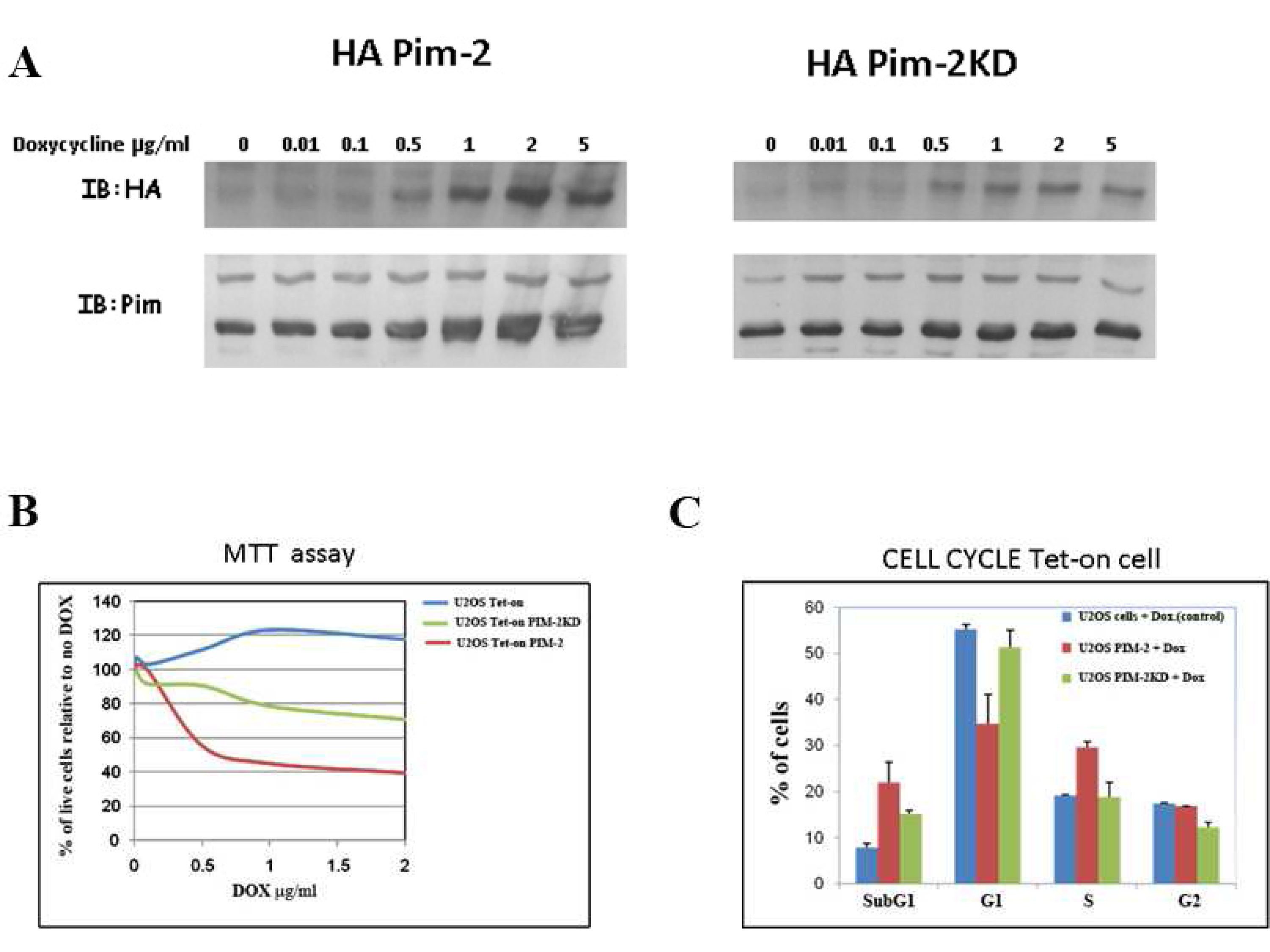

Supplement: Figure S5 — Doxycycline dose-dependent expression of either HA-PIM-2 or HA-PIM-2KD in a stable Tet-on-inducible system in U2OS cells. (A) Total protein extracts from cultures treated with the indicated concentrations of doxycyclin, were analyzed by Western blotting using anti-HA and anti-PIM-2 antibodies for detection of the recombinant proteins (IB:HA and IB:Pim, respectively). (B) Survival of U2OS Tet-on cells expressing either HA-PIM-2 (U2OS Tet-on Pim-2), or HA-PIM-2-Kinase Dead (U2OS Tet-on Pim-2KD), 96 h after activation of expression by increasing concentrations of Doxycycline, as indicated. Survival rates were determined by the MTT assay, compared to cells not treated with Doxycycline. Tet-on U2OS cells with no Pim-2 constructs were used as control (U2OS Tet-on). This panel represents an experiment that was executed in quadruplicates with very small standard error values. (C) Percent cells at the different phases of the cell cycle, as determined by FACS analysis. All cells (as indicated in panel B) were exposed to Doxycycline (2 µg/ml) for 96 h. (TIF) [file pone.0034736.s005.tif]

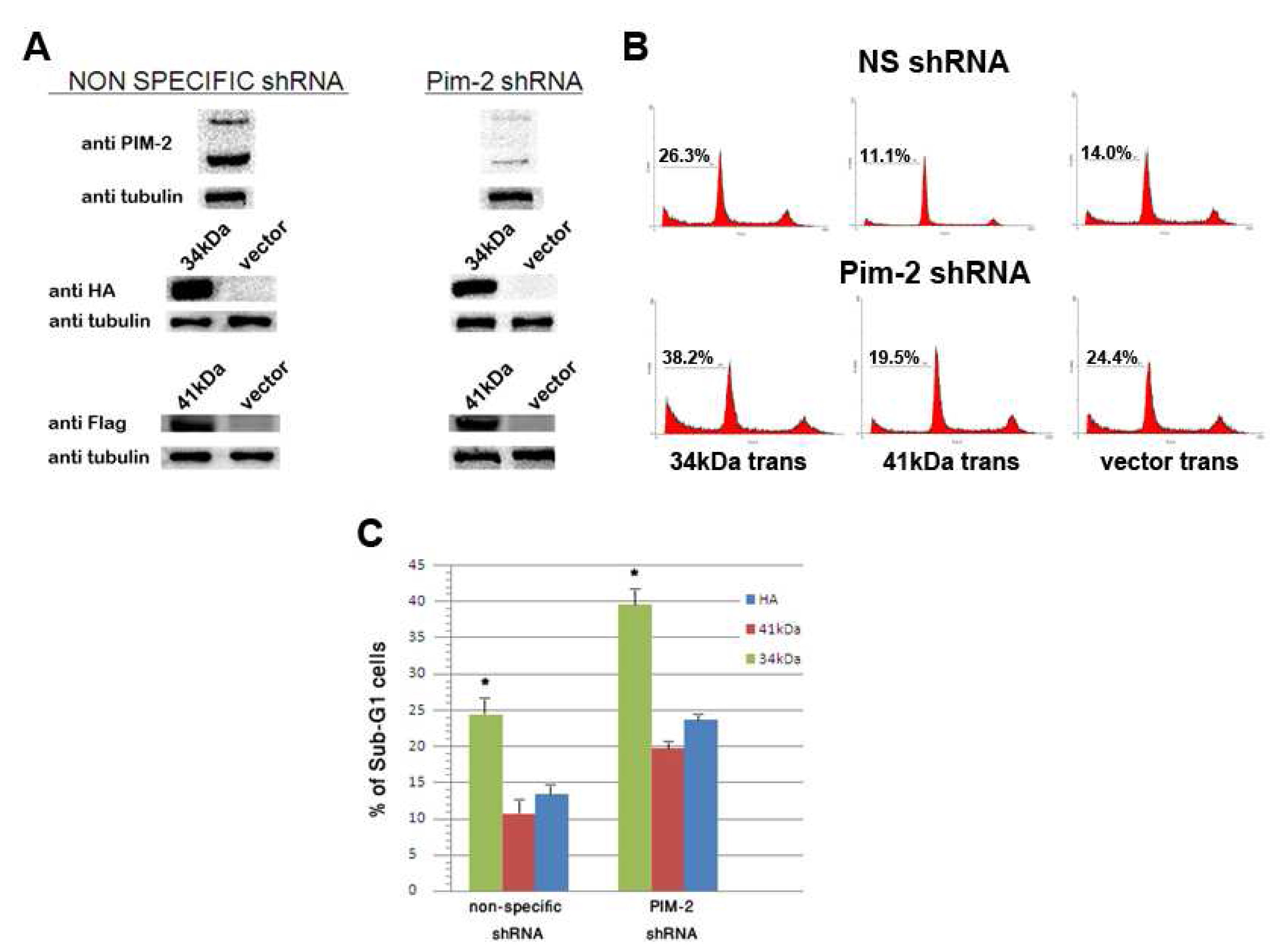

Supplement: Figure S6 — Differential effects of over-expressing either the 34 kDa or 41 kDa isoformes in endogenous PIM-2 silenced cells. (A) Western blots showing silencing of endogenous PIM-2 in U2OS cells, using anti-PIM-2 antibodies (upper panel), over-expression of HA-tagged 34 kDa isoform using anti-HA antibodies (middle panel), and over-expression of Flag-tagged 41 kDa isoform using anti-Flag antibodies (lower panel). Blots were stripped and reprobed with anti-tubulin antibodies for equal loading assessment. (B) Sub-G1 analysis of U2OS cells treated with either PIM-2 shRNA or non-specific (NS) shRNA as control, each transfected with either the HA-tagged 34 kDa encoding plasmid, the Flag-tagged 41 kDa encoding plasmid, or with an empty HA vector as control. Percent of cells at the sub-G1 phase is indicated in each panel. (C) Average percentage of cells (treated as described in panel B) in sub-G1 phase. Asterisks represent statistically significant differences (p<0.05). (TIF) [file pone.0034736.s006.tif]

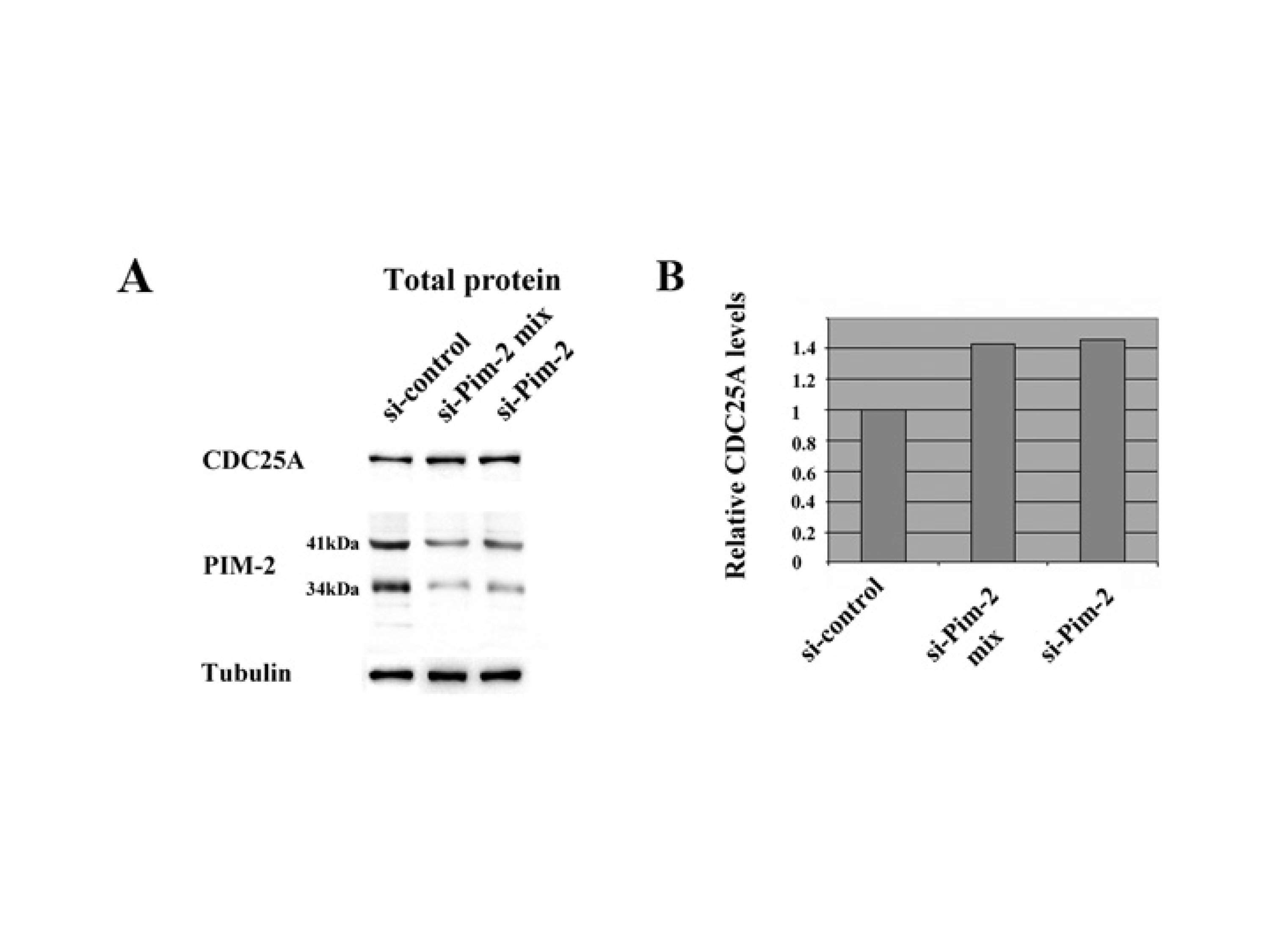

Supplement: Figure S7 — Analysis of CDC25A levels in PIM-2 silenced cells. CDC25A levels were increased by about 40% in PIM-2 silenced cells in two independent experiments using two different sets of siRNS oligos to silent PIM-2 (Ambion – si-Pim-2 or Sigma – si-Pim-2 mix). Scrambled si-RNAs were used for control experiments. Tubulin antibody was used as a control for equal protein loading and served as reference for densitometric analysis. (TIF) [file pone.0034736.s007.tif]
